# Long noncoding RNAs in pancreas cancer: from biomarkers to therapeutic targets

**DOI:** 10.55730/1300-0144.5724

**Published:** 2023-09-09

**Authors:** Esra GÜZEL TANOĞLU, Seyfure ADIGÜZEL, Alpaslan TANOĞLU, Zehra Betül AYDIN, Gülizar HOCAOĞLU, Samet EBİNÇ

**Affiliations:** 1 Department of Molecular Biology and Genetics, Hamidiye Institute of Health Sciences, University of Health Sciences, İstanbul, Turkiye; 2 Experimental Medicine Research and Application Center, University of Health Sciences, İstanbul, Turkiye; 3 Department of Internal Medicine, Division of Gastroenterology, School of Medicine, Bahçeşehir University, İstanbul, Turkiye

**Keywords:** Long noncoding RNA, cancer, pancreas, pancreas cancer, biomarker

## Abstract

Long noncoding RNAs (lncRNAs) are noncoding RNA molecules with a heterogeneous structure consisting of 200 or more nucleotides. Because these noncoding RNAs are transcribed by RNA polymerase II, they have properties similar to messenger RNA (mRNA). Contrary to popular belief, the term “ncRNA” originated before the discovery of microRNAs. LncRNA genes are more numerous than protein-coding genes. They are the focus of current molecular research because of their pivotal roles in cancer-related processes such as cell proliferation, differentiation, and migration. The incidence of pancreatic cancer (PC) is increasing around the world and research on the molecular aspects of PC are growing. In this review, it is aimed to provide critical information about lncRNAs in PC, including the biological and oncological behaviors of lncRNAs in PC and their potential application in therapeutic strategies and as diagnostic tumor markers.

## 1. Introduction

Gene expression is an important step in the regulation of changes that occur during vital processes in cells and tissues. Genomic DNA is located in the nucleus of the cell and guides the correct processing of transcription for messenger RNAs (mRNAs). It then migrates to the cytoplasm and initiates the translation of proteins. In order to secure optimal processing, non-protein-coding RNAs are needed. In general, non-protein-coding RNAs are divided into RNA types such as small nuclear RNA (snRNA), carrier RNA (tRNA), and ribosomal RNA (rRNA), which have different end functions [1].

Long noncoding RNAs (lncRNAs) in the genome are responsible for gene regulation and the development of events in the cell, so they have many pivotal roles in the development of diseases. In addition, they undertake different functions in the cell, such as regulating chromatin remodeling, secondary structures attached to proteins, and epigenetic, transcriptional, and translational events [2,3].

The term “lncRNA” originated before the discovery of microRNA. The first lncRNA found in mammalian cells reported in the 1990s was H19. Not long after that discovery, another lncRNA was discovered: the X-inactive-specific transcript (XIST) gene, shown to be critical in X chromosome inactivation. The number of lncRNA genes was found to be higher than the number of protein-coding genes. They are noncoding RNA molecules with heterogeneous structures, usually consisting of 200 nucleotides or more. Because they are transcribed by RNA polymerase II, they have properties similar to mRNA [4,5]. However, they differ from mRNA in terms of their structure as they contain fewer exons, have lower expression levels in different tissues, and contain an open reading frame [6].

Transcribed lncRNAs mature by undergoing certain transcriptional changes. Thus, each lncRNA follows its own unique functional structure. Although they do not code for proteins, most of the intronic and antisense lncRNAs are located in the nucleus and cytoplasm. This shows that transcripts are involved in the regulation of cytoplasmic processes. More than 80% of lncRNAs are found in the nucleus [7]. One of their most well-known functions in the nucleus is to regulate gene and genome activity. LncRNAs are involved in many cellular events such as chromosome rearrangement, histone modifications, modification of genes by alternative splicing, and regulation of gene expression. Cytoplasmic noncoding lncRNAs can act as templates for the synthesis of small peptides with microRNA-like behavior. They can perform mRNA degradation or regulate the translation process [8]. LncRNAs cause genomic changes in many different cancer types and are used for diagnosis and therapeutic treatment in cancer [9].

In this review, the aim is to summarize the expression patterns, biological functions, and molecular mechanisms of lncRNAs that play roles in pancreatic cancer (PC) progression and the potential clinical utility of lncRNAs in PC.

## 2. Pancreatic cancer

The incidence of PC is increasing day by day around the world. This insidious cancer has a 5-year survival rate ranging from 2% to 9%. The mortality rate increases with age and the disease has particularly poor prognosis in men. The formation mechanisms of PC have not been sufficiently elucidated yet. Tobacco use, chronic pancreatitis, diabetes, and genetic factors are known to be important factors for cancer growth [10]. In recent years, scientific studies have focused on the link between chronic pancreatitis and the development of PC. It is thought that the progression to PC can be clarified by deciphering the molecular codes of chronic pancreatitis [11,12].

Unfortunately, PC has limited treatment options. Therefore, elucidating molecular tumor mechanisms in a detailed manner will have indispensable effects in terms of follow-up and treatment. Although genetic mutations such as *ATM*, *BRCA1/2*, and *PALB2* are effective in PC initiation and progression, epigenetic factors also play pivotal roles [13]. The most common genetic mutations in PC include p53 [8], *CDKN2A* [9], *SMAD4* [10], and *KRAS* mutations [14]. In this hereditary cancer, mutations in the *BRCA2*, *STK11*, *ATM*, *PALB1*, *MLH1*, *BRCA1*, *TP53*, *MSH2*, and *CDKN2A* genes also significantly affect the familial PC history [15]. Surgery, chemotherapy, and radiotherapy strategies are at the forefront considering current treatment methods. The elucidation of the molecular features of cancer increases the success of targeted personalized therapy. Systemic chemotherapy combinations containing 5-fluorouracil, folinic acid (leucovorin), irinotecan, and oxaliplatin (FOLFIRINOX) and gemcitabine plus nab-paclitaxel are frequently used as treatment regimens for patients with advanced PC. Conventional chemotherapy agents are used in a large number of PC cases [16]. However, clinical applications are limited due to low selectivity and numerous systemic side effects. In fact, the ability of chemotherapy to induce cancer cell death is due to its strong cytotoxicity, inhibiting the process of cell division and mitosis [17]. Biomarker-based research is ongoing to develop potential drug targets for PC therapy. The impact of genetic markers for microRNAs (miRNAs) or lncRNAs interacting with protein-coding genes is a focus for researchers in studies of pancreatic ductal adenocarcinoma (PDAC) [18]. Studies performed in previous years showed that lncRNAs play a central role in cancer biology. Researchers continue to show that lncRNAs are specifically expressed or deregulated in many cancer types; hence, lncRNAs are used as biomarkers. In addition, it was shown that lncRNAs can be used for the determination of cancer treatment response. In addition, lncRNAs play roles in the regulation of epigenetic modification of the cancer process, alternative splicing, transcription, and translation mechanisms. As lncRNAs have roles in regulating the biological behavior of cancer cells, they also affect events such as epithelial–mesenchymal transition (EMT), migration, proliferation, and chemoresistance [19].

## 3. LncRNAs in cancer

LncRNAs are involved in the cell cycle, proliferation, differentiation, metabolism, apoptosis, and maintenance of pluripotency. In other words, lncRNAs play active roles in the regulation of many processes [20]. LncRNAs have varying expression levels in different cell types. LncRNAs function as transcriptional factors. It was reported that lncRNAs cause changes in transcription and translational levels, and they also cause tumorigenesis. Abnormal levels of lncRNA expression play a role in cancer development and progression, and lncRNA expression is important in therapeutic treatment as it has the potential to be a biomarker for many diseases. Very few lncRNAs circulate from the cytoplasm and can be isolated from any bodily fluids, tissues, or cells [21]. They generally have oncogenic properties in cancer and little functionality as tumor suppressors. However, lncRNAs were found to function as both oncogenes and tumor suppressors. Overexpression of PCA3, one of the lncRNAs, was the first widely used biomarker in prostate cancer. In urine samples, PCA3 was reported to have specificity of 59%–76% and sensitivity of 58%–82% [22,23].

It was postulated that the overexpression of HOX antisense intergenic RNA (HOTAIR) is important for many types of cancers. HOTAIR is overexpressed in solid tumors in particular, acting as both an oncogene and a tumor suppressor. It plays an inevitable role in the shaping of cancer events such as tumor growth, invasion, and metastasis, and it is linked to poor prognosis [24]. It was reported that HOTAIR is deregulated in hepatocellular and colorectal carcinomas, pancreatic tumors, and tumors with poor prognosis such as ovarian cancer. In esophageal cancer, increased expression of HOTAIR facilitates EMT and promotes metastasis and invasion. Moreover, it was shown that increased HOTAIR expression in bladder transitional cell carcinoma causes poor prognosis [25]. A study of tissues from 300 patients with gastric cancer showed that increased HOTAIR expression was associated with peritoneal cancer diffusion [26].

Metastasis-related lung adenocarcinoma transcript 1 (MALAT1), located on chromosome 11q13.1, is involved in gene regulation at the transcriptional and posttranscriptional levels. MALAT1 modulates the activity of the spliceosome complex, which is necessary for the correct addition and activity of B related to the transcriptional factor Myp (B-Myp) during the transition to the G2/M mitotic phase. Increased upregulation of MALAT1 was observed in lung and bladder cancers, esophageal squamous cell carcinoma, and glioma [27]. MALAT1 was suggested for use as a biomarker in the early diagnosis of prostate cancer. Patent applications were filed by some researchers for the use of HOTAIR and MALAT1 (CN105586399A) as adjunctive biomarkers in gastric cancer. In a study of gastric cancer, it was found that high expression of MALAT1 resulted in lower survival rates compared to the normal group. It was also shown that MALAT1 might be a biomarker for the recurrence potential of hepatocellular carcinoma after liver transplantation. MALAT1 was reported to increase invasion and metastasis in breast cancer cells [13,28].

**Figure 1:**
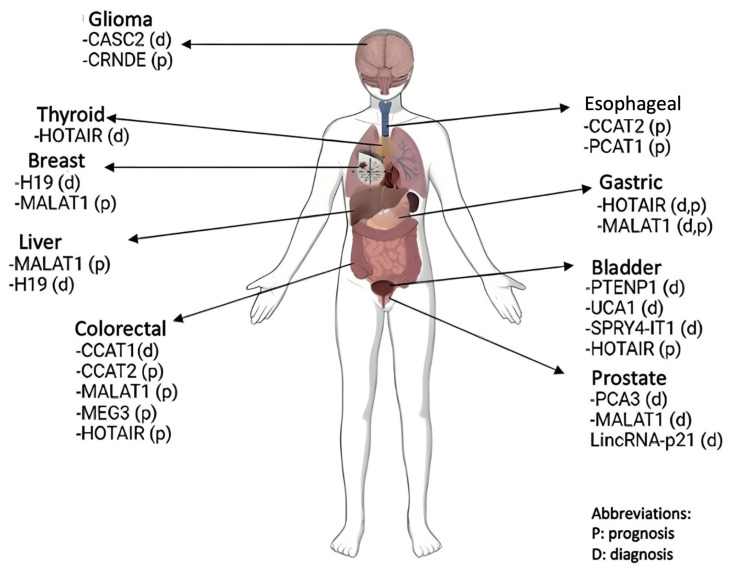
LncRNAs that play roles in diagnosis and prognosis in different tissues.

It was shown that the expression level of NEAT1, a type of lncRNA transcribed from the multiple endocrine neoplasia locus, is increased in solid tumors. However, it has decreased expression levels in cases of leukemia and multiple myeloma. NEAT1 expression varies according to the cell in which it is located, and it has potential as a biomarker and can be targeted therapeutically. Expression changes in tumor tissues provide information about the prognosis of cancer [29]. Some tumor-suppressor lncRNAs, such as MEG3, GAS5, neuroblastoma-associated transcript-1 (NBAT-1), and long intergenic noncoding RNA/p53-induced transcript (LINC-PINT), have decreased expression levels in cancer cells [30]. Among them, MEG3 expression is epigenetically altered. It was determined that the expression level of MEG3 is considerably reduced in brain, lung, colon, and liver cancers and leukemia. MEG3 works together with different miRNAs to induce apoptosis in tumors. It regulates the TGF-beta genes that affect invasion and the immune system in cancer. In addition, it activates an important target, p53 protein [31]. H19 has high specificity and high sensitivity for expression in the cell in cancer. Plasma H19 expression levels have higher sensitivity than conventionally used biomarkers in breast cancer patients. Working with many miRNAs, H19/miR-675 activates EGFR and c-Met [32]. The expressions of some lncRNAs that vary according to tissues show high specificity, sensitivity, and accuracy when these lncRNAs are evaluated together with helper biomarkers. Examples of the use of lncRNAs for both diagnosis and prognosis in the early diagnosis and treatment of various types of cancer were reported [33,34]. LncRNAs that play roles in diagnosis and prognosis in different tissues are given in Figure 1.

## 4. LncRNAs in pancreas cancer

### 4.1. Oncogenic and tumor-suppressor roles of lncRNAs in PC

LncRNAs affect various behaviors of pancreatic malignant cells such as the proliferation, differentiation, or migration of tumor cells. LncRNAs are a focus for molecular research due to their effects on cancer-related processes such as cell proliferation and migration [35].

There is increasing interest in the effects of lncRNAs in PC tissues. The lncRNA most highly expressed in PC was identified as MACC1-AS1 and it is particularly expressed in patients with low survival. MACC1-AS1 increases the expression of the PAX8 protein, which plays a role in aerobic glycolysis, and promotes the proliferation and metastasis of PC cells by activating NOTCH1 signaling. The MACC1-AS1/PAX8/NOTCH1 signaling axis was proposed as a therapeutic target for the treatment of PC in the literature [36].

It was reported that HOTAIR functions as an oncogenic lncRNA in PC. HOTAIR, a HOX antisense intergenic RNA, combines with PRC2 (polycomb repressive complex 2) to transcriptionally silence the HOXD locus. With the increased regulation of HOTAIR, the cell cycle progresses, cell proliferation is ensured, and apoptosis of cancer cells is inhibited. In vitro experiments suggested that HOTAIR influences cell proliferation and regulates apoptosis [37], which are required for cancer growth.

Another lncRNA associated with the HOX gene is HOXA transcript at the distal tip (HOTTIP). HOTTIP binds to the WDR5 protein and activates HOXA transcription, causing H3K4 methylation. Among the genes targeted by HOTTIP, aurora kinase A (AURKA) was reported to inhibit apoptosis, regulate cell growth, and induce cell migration independently of WDR5 [38]. The lncRNA FGD5 antisense RNA 1 (FGD5 AS1) was found to be highly expressed in PC and plays a role in cell proliferation, migration, and invasion. In the same study, it was shown that FGD5-AS1 activates the Wnt/β-catenin signaling pathway by suppressing miR-577, a tumor suppressor in PC. It was reported that the expression of NUTF2P3-001, another lncRNA that increases in cases of hypoxia, plays a positive role in the regulation of KRAS expression and supports cell proliferation in PC [39].

It was shown that NR2F1-AS1 plays a role as a carcinogenic factor in various cancer types and is associated with poor prognosis. In PC, NR2F1-AS1 is associated with cell proliferation, cell migration, and metastasis, and these effects are manifested by hypoxia-inducible factor-1α (HIF-1a). Therefore, NR2F1-AS1 was found to be a hypoxia-inducible lncRNA. The AKT/mTOR signaling pathway is activated by increased regulation of NR2F1-AS1 in hypoxic environments. The active AKT/mTOR pathway promotes PC cell proliferation, migration, and invasion. Moreover, it was suggested that NR2F1-AS1 may be a potential prognostic biomarker and therapeutic target for PC [40].

The lncRNA LINC00941 was reported to play an oncogenic role in proliferation and metastasis in PC. The increase in ROCK1 expression initiates LIMK1/cofilin-1 signaling, which promotes tumorigenic activities. These results demonstrate the potential for LINC00941 to be a diagnostic biomarker in PC [41].

CERS6-AS1 activates the ERK signaling pathway by binding to miR-217 to regulate YWHAG, which is involved in the phosphorylation of RAF1. miR-217 suppresses cell proliferation and metastasis by directly targeting YWHAG. The binding of CERS6-AS1 to miR-217 activates the ERK signaling pathway, thus promoting cell proliferation, cell migration, and metastasis [42]. Hence, the CERS6-AS1/miR-217/YWHAG/RAF1 signaling axis has the potential to be a therapeutic target for PC [43].

A recent study found that the expression of the lncRNA BM466146.1 was significantly reduced in PC tissues compared to normal pancreatic tissues. Because BM466146.1 regulates transcription as a transcription inhibitor of its neighboring gene, zinc finger protein 24 (ZNF24), it was named ZNF24 transcription regulator (ZNFTR). ZNFTR was found to play a role in inhibiting the proliferative, metastatic, and proangiogenic properties of PC cells. In addition, it was shown that low expression rates of ZNFTR, which functions as an inhibitor, are associated with low survival rates. Another lncRNA, LINC00337, acts as an E2F1 coactivator in PC, increasing the expression of target proteins and promoting cell proliferation [44].

The lncRNA GAS5 has antiproliferative effects in PC cells. The expression level of GAS5, which is decreased in PC, increases PC cell proliferation by negatively regulating the expression of cyclin-dependent kinase 6 (CDK6). Methylation-mediated LINC00261 suppresses c-Myc transcription. Thus, it inhibits cell proliferation, migration, and metastasis in PC [45].

Knockdown of the lncRNAs LINC01559 and UNC5B-AS1 was found to cause decreased glucose uptake and lactate production in PC cells. Thus, they play a role in suppressing tumor growth by reducing the glycolytic capacity of PC cells [46].

LINC00976 expression was found to be increased in PC, and it was associated with poor prognosis. It was reported that when LINC00976 is silenced, cell proliferation and invasion are also suppressed. LINC00976 targets OTUD7B, which deubiquitinates EGFR, thereby activating the MAPK signal [47].

LncRNAs have tumor-suppressive properties as well as oncogenic and therapeutic potential. With these properties, lncRNAs play an important role in the formation and development of human cancers [48].

In a recent study, overexpression of LINC01963 inhibited proliferation and invasion in PC cells and also induced apoptosis [49]. Another lncRNA, MEG3, was shown to suppress PC progression by regulating the activity of apoptotic signal sequences. At the same time, it was determined that patients with low MEG3 expression rates had poor prognosis in survival analyses [50].

LINC00261 was found to be downregulated in PC tissues and stem cells. Likewise, ITIH5 was shown to be downregulated in PC cells. In the presence of LINC00261, the expression of ITIH5 is increased, thus increasing the sensitivity of PC stem cells to gemcitabine while reducing tumorigenic properties such as cell proliferation and invasion [51].

FLVCR1-AS1 is an important regulator involved in cancer progression. Lin et al. reported that FLVCR1-AS1 is downregulated in PC and is associated with poor prognosis. FLVCR1-AS1 overexpression was shown to suppress cell proliferation, cell cycle, and migration by activating PTEN/AKT signaling. It was reported that LINC01111 can suppress the metastatic ability of neoplastic cells in PC while lncRNA GAS5 suppresses PC metastasis by regulating the miR-32-5p/PTEN axis [52]. LncRNAs related to PC are given in the Table.

### 4.2. LncRNAs that play important roles in invasion and metastasis in PC

In PC, lncRNAs regulate important biological events such as the proliferation, invasion, and metastasis of cells [53]. Overexpression of LINC01232 in PC causes increased upregulation of HNRNPA2B1. HNRNPA2B1 promotes PC metastasis by playing a role in the alternative splicing of A-Raf, the protein kinase in the MAPK signaling pathway. Therefore, LINC01232 plays an active role in the metastasis of PC [54]. It was reported that overexpression of HOTAIR may increase cell invasion in PC cell lines. When HOTTIP, another lncRNA, was silenced in PC, it was observed that migration in PC cells decreased [55].

In a recent study, LncRNA LOC389641 expression was significantly increased in PDAC tissues. It was stated that LOC389641 contributes to EMT by increasing Vimentin and Snail expression and suppressing E-cadherin expression [56].

Sun et al. reported that decreased lncRNA ENST00000480739 expression levels in PC tissues are effective in cancer metastasis. In in vitro experiments, increased expression of ENST00000480739 directly targeted osteosarcoma amplified-9 (OS-9) and led to its upregulation. OS-9 is effective in suppressing invasion and metastasis by interacting with HIF-1 and HIF-1a. Overexpression of HIF-1 and HIF-1a in PC was suggested to play a critical role in invasion and metastasis. Therefore, the production of drugs targeting the lncRNA ENST00000480739/OS-9/HIF-1 signaling pathway may be promising for the treatment of PC [57]. In patients with PDAC, patients with lymph node metastases were found to have lower expression levels of ENST00000480739 than patients without metastasis. Thus, ENST00000480739 was shown to have the potential to be a new biomarker to evaluate metastasis status [57]. LncRNA H19 is an important maternally inherited oncogenic factor. It is effective in the malignancy of tumors by affecting specific miRNAs in many cancer types, such as PDAC [58–60]. H19 expression is significantly increased in metastatic PDAC. It was found that invasion and metastasis were inhibited when H19 was silenced in PDAC cells [61].

In a study by Wang et al., miR-181a was downregulated and ANRIL was upregulated in PC tissues. Downregulated miR-181a was also found to increase HMGB1 expression by targeting HMGB1, which is involved in the activation of cell autophagy. In summary, ANRIL activates HMGB1-induced cell autophagy by targeting miR-181a. Overexpression of ANRIL promotes cell proliferation, invasion, and migration in PC cells and increases their resistance to gemcitabine. Likewise, with the degradation of ANRIL, cell proliferation, migration, invasion, and resistance to gemcitabine are suppressed. As a result of this study, it was suggested that ANRIL and miR-181a may be potential targets for PC therapy [41].

LncRNA BX111, which is overexpressed in PC tissues, plays a role in lymphatic vessel invasion and distant metastasis, leading to a decrease in patient survival rates [62]. There is a correlation between high expression levels of HOTTIP in PC and survival rates [63].

5-Methylcytosine (m5C) methylation is a posttranscriptional modification that plays an important role in RNA metabolism. It was shown that m5C methyltransferases play a role in cell proliferation in many cancers. While m5C regions are known to be abundant in lncRNAs, their exact functions are not known. In PC, AC009974.1 was found to be effective in the EMT process [64].

Increased expression of LINC00462 contributes to the invasion and metastasis processes of PC by accelerating the EMT process. On the other hand, LINC00462, LINC00958, SNHG12, and OIP5-AS1 are important lncRNAs involved in the progression of EMT in PC [65].

It was shown that CASC9 promotes PC progression and invasion by interacting with miR-497-5p and also affect Cyclin D1 [66].

MALAT-1 is overexpressed in PC stem cells and plays a role in angiogenesis and proliferation. It is also effective in increasing resistance to drugs during the treatment of PC. The expression of MALAT-1 is also effective during the EMT process in PC cell lines. With the suppression of MALAT-1 expression, it was observed that the expression levels of N-cadherin and Vimentin were decreased and the expression of E-cadherin was increased [67]. In PC, MALAT-1 also decreased the expression of Sox2. It was reported that MALAT-1 can accelerate the proliferation and metastasis of PC cells by stimulating autophagy [68]. Another lncRNA, ROR, contributes to EMT in PC by causing inhibition of p53 and ZEB1 expression [69].

### 4.3. LncRNAs in the apoptosis of pancreatic cancer cells

It was observed that PC cells undergo apoptosis with the degradation of HOTAIR and HOTTIP. Silencing of HOTAIR in Panc1 cells reduced the interaction of the histone-lysine N-methyltransferase enzyme, EZH2, with the promoter region of proapoptotic gene GDF15. The suppression of GDF15 also supports the apoptosis of tumor cells [70]. AF339813, a lncRNA upregulated in PC, was also reported to induce apoptosis via mitochondria and caspase-dependent pathways [71].

A recent study reported that GATA3 AS1 knockdown reduces the cell proliferation and invasion capabilities of PANC 1 or AsPC 1 cell lines while increasing cell apoptosis. GATA3-AS1 was reported to modulate the Wnt/β-catenin pathway in association with miR-30b-5p/Tex10 and it regulates cell proliferation, invasion, and apoptosis processes. As a result of this study, it was suggested that the GATA3-AS1/miR-30b-5p/Tex10 signaling axis could be used in the diagnosis and treatment of PC [72].

CERS6-AS1 was found to be highly upregulated in PC cells in a recent study. When CERS6-AS1 is silenced, it suppresses the proliferation of PC cells and increases cell apoptosis. CERS6-AS1 interacts with miR-195-5p, increasing the expression of WD repeat domain phosphoinositide interacting 2 (WIPI2-WD repeat domain phosphoinositide interacting 2). Upregulation of WIPI2 inhibits apoptosis and increases cell proliferation [73].

CTD-3252C9.4 was reported to be downregulated in PC cells and tissues. Overexpression of CTD-3252C9.4 suppressed cell proliferation, migration, and invasion while increasing apoptosis. The antiproliferative and proapoptotic effects of CTD-3252C9.4 are mediated by downregulation of IFI6. Overexpression of IFI6 has tumorigenic effects. IFI6 is targeted and downregulated by CTD-3252C9.4. IRF1 is blocked by CTD-3252C9.4 to inhibit IFI6 transcription [74].

**Figure 2:**
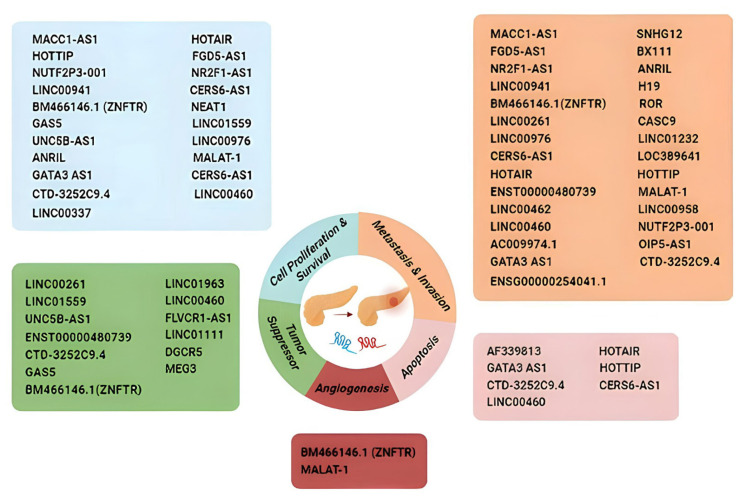
LncRNAs that are effective in cancer progression.

A recent study found that LINC00460 knockdown inhibits cell proliferation, migration, and invasion and promotes apoptosis. LINC00460 was able to directly target miR-320b, and downregulation of LINC00460 significantly increased the miR-320b level. Previous studies showed that miR-320 has a suppressive effect on increased migration and invasion in proliferation, while, on the contrary, downregulation of miR-320b contributes to invasion and EMT. This recent study revealed the effect of miR-320, which is increased by LINC00460 degradation, on proliferation, migration, and apoptosis in PC [75]. LncRNAs that are effective in cancer progression are summarized in Figure 2.

### 4.4. LncRNAs in drug resistance

Oncogenic lncRNAs such as LINC00346, linc-ROR, TUG1, and AB209630 are associated with gemcitabine resistance in PC. For example, knockdown of linc-ROR can reduce gemcitabine resistance by targeting HOXA13 [76]. Unlike others, overexpression of lncRNA AB209630 was reported to suppress gemcitabine resistance. The overexpression of AB209630 inhibits the PI3K/AKT signaling pathway and suppresses drug resistance. Another tumor suppressor in PC, MEG3, was also reported to suppress gemcitabine resistance [77].

Yin et al. reported that HOTTIP plays a role in cisplatin resistance by suppressing miR-137 expression [78]. In a recent study, HIF1a antisense RNA1 (HIF1A-AS1) expression levels were significantly increased in gemcitabine-resistant PC cells. As a result of that experiment, increased HIF1A-AS1 expression was shown to upregulate HIF1a to support glycolysis. Thus, overexpression of HIF1A-AS1 was found to increase gemcitabine resistance in PC and cause a decrease in survival [79].

UPK1A-AS1 induced by IL8/NF-kappa B signaling was found to be a lncRNA involved in drug resistance in PC. The blockade of UPK1A-AS1 expression increased the oxaliplatin sensitivity of tumor cells. UPK1A-AS1 is induced by IL-8/NF-kappa B signaling. UPK1A-AS1 enables DNA double-strand break repair by reinforcing the interaction between Ku70 and Ku80 in nonhomologous end-joining (NHEJ) repair. UPK1A-AS1 expression appeared to be associated with poorer chemotherapeutic response and shorter survival time in PDAC patients. As a result of this study, it was concluded that UPK1A-AS can be used as a biomarker to observe the response to platinum-based chemotherapy in patients with PDAC [80].

### 4.5. LncRNAs as biomarkers in pancreatic cancer

Studies have shown that lncRNAs have extensive functions in mediating PC progression and may therefore serve as prognostic markers or therapeutic targets [13,19,20]. In a recent study, it was suggested that the expression levels of PVT1 and HIF-1a could be used as biomarkers for survival in PC patients [46].

Overexpression of LINC00675 was associated with progression of lymph node metastasis and perineural invasion in PC. It was reported that LINC00675 can be used as a marker for the prediction of disease recurrence after surgical resection in PDAC [81].

It was also suggested that highly regulated C9orf139 could be a biomarker for determining PC staging [82]. Overexpression of RUNX1-IT1 was identified to be a factor that increases mortality, and it was reported to be a biomarker showing poor survival. Increased expression of LINC01963 and LINC00261 was shown to be significantly associated with higher survival rates in PC patients. Increased expression levels of MACC1-AS1, LINC00462, LINC01559, and UCA1 were associated with shorter survival rates [65]. It was also suggested that lncRNAs such as UFC1, RP11-263F15.1, ABHD11-AS1, and HULC can be used as diagnostic markers in PC [65]. The HOTAIR-miR-613-Notch3 signaling axis is a promising therapeutic target for PC [83]. It is important to identify the expression profiles of lncRNAs in order to discover biomarkers that will enable the diagnosis and treatment of PC in the early stages with noninvasive methods.

## 5. Conclusion

LncRNAs can be used as biomarkers in cancer cases as well as to predict prognosis. These molecules also play leading roles in the regulation of the genes they target by interacting with various cell signaling pathways, but, as a challenge, they are affected by various factors such as age, sex, nutrition, and hormones [84,85]. On the other hand, the aberrant expressions and single nucleotide polymorphisms of some lncRNAs that play roles as suppressors of protooncogenes and tumors are associated with tumorigenesis and metastasis. Therefore, lncRNAs hold strong promise for cancer diagnosis and treatment. However, there are still controversial gray areas and limitations in this regard. First of all, many lncRNAs have been discovered, but their use in clinical practice will not be easy without demonstrating their clear links to particular cancer types or subtypes. Secondly, lncRNAs are a relatively new topic in cancer research, and since the structural and functional information of most lncRNAs cannot be characterized in detail, it is difficult to link lncRNAs with cancer processes [86]. Moreover, without a detailed explanation of the structure and functions of lncRNAs, efforts to develop lncRNA-based therapies seem to be overly optimistic. On the other hand, in order to fully reveal the potential of lncRNAs in cancer diagnosis and targeted therapy, it is necessary to describe each lncRNA in detail and to elucidate its cellular functions and roles in diseases [87].

Finally, chromosome rearrangement, histone modifications, modification of genes by alternative splicing, and regulation of gene expression are all different activities of lncRNAs. Moreover, they can perform mRNA degradation or regulate the translation process. All of these mechanisms are extremely important in the pathophysiology of cancer growth. In other words, lncRNAs can be used for the diagnosis of many types of cancer including PC [88]. In conclusion, lncRNAs can be used as biomarkers and may be applied in therapeutic interventions in cases of PC.

## Figures and Tables

**Table:** Summary of lncRNAs related to pancreatic cancer.

LncRNA	Genomic location	Function	Expression	Mechanism of action
**MACC1-AS1**	7p21.1	Oncogenic	↑	MACC1-AS1 → PAX8 → NOTCH1 → Proliferation, metastasis
**HOTAIR**	12q13.13	Oncogenic	↑	HOTAIR/ PRC2 ⤏ HOXD → Proliferation, invasion, apoptosis inhibition
**HOTTIP**	7p15.2	Oncogenic	↑	HOTTIP/ WDR5 → H3K4me3 at HOXA9 → Proliferation, migration, apoptosis inhibition HOTTIP → HOXA13 → EMT
**FGD5-AS1**	3p25.1	Oncogenic	↑	FGD5-AS1 ⤏ miR-577 → Wnt/ β Katenin → Proliferation, migration, invasion
**NUTF2P3-001**	9q21.2	Oncogenic	↑	NUTF2P3-001→ KRAS → ProliferationHIF-I*a* → NUTF2P3-001 → Invasion
**NR2F1-AS1**	5q15	Oncogenic	↑	NR2F1-AS1 → NR2F1→ AKT/mTOR → Proliferation, migration, invasion
**LINC00941**	12p11.21	Oncogenic	↑	LINC00941 ⤏ miR-335-5p →ROCK1 → LIMK1/Cofilin-1 → Proliferation, metastasis
**CERS6-AS1**	2q24.3	Oncogenic	↑	CERS6-AS1 ⤏ miR-217 → YWHAG → ERK→ Proliferation, migration, metastasisCERS6-AS1 ⤏ miR-195-5p → WIPI2 → reduced proliferation, inhibit apoptosis
**BM466146.1 (ZNFTR)**	18:35,446,176–35,446,941	Oncogenic	↓	BM466146.1 → Poor survival time, poor prognosis
**BM466146.1 (ZNFTR)**	18:35,446,176–35,446,941	Tumor supressive	↑	BM466146.1 (ZNFTR) ⤏ ATF3 → ZNF24 ⤏ VEGFA → inhibit proliferative, metastatic, pro-angiogenic capacities
**NEAT1**	11q13.1	Oncogenic	↑	NEAT1 ⤏ miR-506-3p → Proliferation
**LINC00337**	1p36.31	Oncogenic	↑	LINC00337 → E2F1 → Proliferation, regulate cell cycle
**LINC00261**	20p11.21	Tumor supressive	↑	LINC00261 → p300/CBP ⤏ c-Myc → Inhibit proliferation, migration, metastasisLINC00261 → ITIH5/GATA6 → Reduced proliferation, invasion and increased sensitivity of gemsitabine
**GAS5**	1q25.1	Tumor supressive	↑	GAS5 ⤏ miR-32-5p → PTEN → supresses metastasis
**GAS5**	1q25.1	Oncogenic	↓	GAS5 ⤏ CDK6 → Poliferation
**LINC01559**	12p13.1	Tumor supressive	↓	LINC01559 → inhibit aerobic glycolysis
**UNC5B-AS1**	10q22.1	Tumor supressive	↓	UNC5B-AS1 → inhibit aerobic glycolysis
**LINC00976**	8q24.21	Oncogenic	↑	LINC00976 → OTUD7B/EGFR → MAPK → Proliferation, invasion
**LINC01963**	2q35	Tumor supressive	↑	LINC01963 → Inhibit proliferation, invasion, induced apoptosis
**MEG3**	14q32.2	Tumor supressive	↑	MEG3 → PI3K/AKT/Bcl-2/Bax/siklin D1/P53 → Suppresses tumor progressionMEG3 → PI3K/AKT/MMP-2/MMP-9 → Suppresses tumor progression
**FLVCR1-AS1**	1q32.3	Tumor supressive	↑	FLVCR1-AS1 → PTEN/AKT → Suppresses proliferation, migration
**LINC01111**	8q21.13	Tumor supressive	↑	LINC01111 → suppresses the metastatic ability of neoplastic cells
**DGCR5**	22q11.21	Tumor supressive	↑	DGCR5 ⤏ miR-27a-3p → BNIP3 → p38 MAPK → apoptosis
**LINC01232**	13q32.3	Oncogenic	↑	LINC01232 → HNRNPA2B/ A-Raf → MAPK → metastasis
**LOC389641**	8p21.3	Oncogenic	↑	LOC389641→ Vimentin/ Snail ⤏ E-cadherin → EMT
**ENST00000480739**	12q13.3	Tumor supressive	↓	ENST00000480739 → OS-9 → HIF-1/ HIF-1*a* → suppresses invasion, metastasis
**H19**	11p15.5	Oncogenic	↑	H19 → Invasion, metastasis
**ANRIL**	9p21.3	Oncogenic	↑	ANRIL ⤏ miR-181a → Proliferation, invasion, migration and increases gemsitabine resistance
**BX111**	10p11.22	Oncogenic	↑	BX111→ metastasis, angiogenesis
**AC009974.1**		Oncogenic	↓	AC009974.1 → EMT
**LINC00462**	13q14.2	Oncogenic	↑	LINC00462 → EMT, invasion, metastasis
**LINC00958**	11p15.3	Oncogenic	↑	LINC00958 → EMT
**SNHG12**	1p35.3	Oncogenic	↑	SNHG12 → EMT
**OIP5-AS1**	15q15.1	Oncogenic	↑	OIP5-AS1 → EMT
**CASC9**	8q21.13	Oncogenic	↑	CASC9 → Glycolysis metabolism ↑ → EMT
**MALAT-1**	11q13.1	Oncogenic	↑	MALAT-1 → Vimentin/ E-cadherin → EMT
**ROR**	18q21.31	Oncogenic	↑	ROR ⤏ p53/ ZEB1 → EMT
**GATA3**-**AS1**	10p14	Oncogenic	↑	GATA3-AS1 ⤏ miR-30b-5p → Tex10 → Wnt/β-catenin → Proliferation, invasion, apoptosis inhibition
**CTD-3252C9.4**	19p13.12	Tumor suppressive	↑	CTD-3252C9.4 ⤏ IFI6 → reduced proliferation, migration, invasion and increased apoptosis
**LINC00460**	13q33.2	Tumor suppressive	↓	LINC00460 → miR-320b → suppresses proliferation, migration, invasion and support apoptosis
